# Patellar and Femoral Bone Morphology Is Associated With Overweight and Sports Participation in Young Adolescents

**DOI:** 10.1002/jor.70089

**Published:** 2025-10-21

**Authors:** Rosemarijn van Paassen, Nazli Tumer, Jukka Hirvasniemi, Tom M. Piscaer, Amir.A. Zadpoor, Stefan Klein, Sita M. A. Bierma‐Zeinstra, Edwin H. G. Oei, Marienke van Middelkoop

**Affiliations:** ^1^ Erasmus MC Medical University Center Rotterdam Rotterdam Netherlands; ^2^ Technical University Delft Delft Netherlands

**Keywords:** anterior knee pain, BMI, Bone shape, femur, knee, patella, patella‐femoral, sports participation, Young adolescence

## Abstract

High levels of physical activity or high BMI during puberty could negatively influence bone and cartilage development. Little is known about the effects of loading on patellar and femoral bone shape in a young population. Therefore, we aim to identify the association between 3D patella and femur shape and biomechanical loading in a young adolescent population. Participants were selected from an ongoing cohort study (Generation‐R study). Participants that underwent knee‐MRI at 13 years‐old follow‐up were included. Patellae and femora were segmented from these MRIs and using these 3D models, statistical shape modeling was performed. Generalized estimating equations were used to analyze the association between loading (BMI, physical activity and sports participation) and shape variation. Bonferroni correction was used to correct for multiple testing. 1912 participants underwent MRI of which 3638 patellae and 3355 femora were included in the statistical shape models. Nine patellar (modes 1–7, 10 and 11) and nine femoral (modes 1–3, 6–10 and 14) shape modes were associated with BMI. Sports participation at thirteen years old was associated with one patellar (mode 1) and two femoral (modes 1 and 6) shape modes. One shape mode (mode 12) was associated with sports participation at 9 and 13 years old. Sports participation and BMI were significantly associated with bone shape variations. BMI was associated with most shape variations found in our statistical shape models, emphasizing the significant impact of BMI on bone morphology during adolescence with implications for musculoskeletal health and injury prevention.

## 1 Introduction

Bone can adapt as a response to mechanical loading [1]. Before puberty, the bone is more responsive to loading and is, therefore, more likely to change to meet biomechanical loading demands than at older ages [2]. Excessive loading during this (pre‐)pubertal period might lead to adaptations in bone shape [3, 4, 5]. During this developmental phase from childhood to adulthood, specific musculoskeletal complaints, such as Osgood‐Schlatter disease (OSD), Sinding‐Larsen‐Johansson syndrome (SLJS), Sever's disease, and patellofemoral pain (PFP) arise [6, 7, 8]. While OSD, SLJS, and Sever's disease are known to be related to skeletal maturation, this relationship is less clear for PFP [6, 7, 8, 9, 10]. As the bone and joint are still developing during puberty, knee alignment, and bone shape may play a role in the development and etiology of different knee complaints and disorders [10, 11, 12]. In adults, several 2‐dimensional (2D) alignment parameters and bone shape measures (e.g., higher Insall‐Salvati (IS) ratio, greater patellar tilt angle, greater bisect offset, and greater sulcus angle) are related to structural abnormalities (e.g., patellar osteophytes, minor cartilage defects, and high fat pad signal) that are associated with patellofemoral joint (PFJ) osteoarthritis (OA) [10].

3‐dimensional (3D) bone shape analysis, using statistical shape modeling (SSM), could provide additional information on bone shape development compared to existing 2D alignment parameters of the joint. 3D SSM provides a more comprehensive understanding of 3D shape features than 2D analyses that are unable to incorporate 3D complexities and can be affected by joint positioning [13]. SSM allows for the assessment of the 3D geometry of bones and provides information on the mean bone shape and shape variation within a study population, which can be used to identify shape‐specific patterns [14]. The available literature on the 3D shape of the PFJ mainly focuses on specific diseases, including PFP and PFOA patients. No previous studies focusing on PFJ shape have yet been performed in a healthy young‐adolescent population in which the skeleton is still developing [13, 15, 16, 17, 18, 19].

High levels and specific types of physical activity in young adolescents can affect the bones and cartilage [20, 21, 22, 23]. Research in the hip showed a relationship between (excessive) loading during growth and the development of cam morphology of the proximal femur in young active soccer players [20, 23, 24, 25]. This specific bone shape variation is also an important risk factor for the development of hip OA later in life, which highlights the importance of better understanding bone shape development [24, 26]. In addition, not only high physical activity levels but also overweight and obesity may cause high loading demands on the patellar and femoral bones [27]. Although there are signs that physical activity and obesity influence bone remodeling during adolescence [5, 28, 29], the exact associations with bone shape development remain unclear.

Given that young‐adolescence is a critical period for skeletal development, gaining more insight into the 3D shape of the knee bones during this period will add to our understanding of the development of knee complaints and disorders during growth and development. Therefore, this study aims to provide a descriptive analysis of knee morphology and its associations with BMI and sports participation, to establish a better understanding of factors influencing adolescent knee health.

## 2 Methods

### 2.1 Study Population

We included a subgroup of participants from the Generation R study, which is a large population‐based cohort study that follows children and their parents from fetal life until adulthood. In total, 9778 pregnant women were included in the study, and their children were born between 2002 and 2006 [30]. The medical ethics committee of the Erasmus MC, University Medical Center Rotterdam, approved all measurements within the Generation R study. Written informed consent was obtained from the parents or caretakers of all children, as well as from children aged 12 and above, as required by Dutch law. Baseline measurements were performed after birth and participants were invited for follow‐up measurements at multiple time points (i.e.,5, 9, and 13 years of age). These follow‐up measurements consisted of two visits to the research center. The first visit included height and weight measurements, while the second included magnetic resonance imaging (MRI). A random subset of the participants underwent MRI of the knee at 13‐year follow‐up. The study population included in this current study consisted of all 1912 participants who underwent MRI of the knee at 13‐year‐old follow‐up. Of these children, data on sports participation at 9‐year‐old follow‐up was also included.

### 2.2 Measurements

Questionnaire data extracted for the current study included demographics (sex and age), physical activity (number of days per week with at least 1 h of physical activity), number and type of sports, and active transportation to and from school (at least one trip a week by foot or bike). Two categorical variables were created, the first one indicated if a child participated in sports at 13 years old. The second variable indicated if a child participated in sports at both 9 and 13 year old or only at 13‐year old follow‐up. Sedentary behavior was measured during weekdays as television viewing (< 1 h/day, 1–2 h/day, and ≥ 2 h/day), computer use (< 1 h/day, 1–2 h/day, and ≥ 2 h/day), and gaming (< 1 h/day, 1–2 h/day, and ≥ 2 h/day).

From the physical measurements, the children's height (stadiometer) and weight (SECA) were used to calculate body mass index (BMI; weight/height^2^ [kg/m^2^]) and standardized BMI scores for age and sex (BMI‐SDS), defined according to the Dutch reference charts [31]. Weight status was classified as underweight, normal weight, and overweight and obesity following the International Obesity Task Force (IOTF) guidelines [32].

### 2.3 MRI Scanning and Post‐Processing of MRI

MRI was conducted on a 3.0 Tesla clinical scanner (Discovery MR750w, GE Healthcare, Milwaukee, WI, USA). A coronal three‐dimensional water‐excitation Gradient Recalled Acquisition in Steady State (3D weGRASS) sequence was employed to acquire high‐resolution isotropic data from both knees. The voxel size was 0.7 × 0.7 × 0.7 mm. Images were subsequently reformatted in both sagittal and axial planes.

An in‐house developed automatic segmentation algorithm was applied to obtain the patellar and femoral bone samples using the 3DGRASS MRIs at 13‐year‐old follow‐up as input [33, 34, 35]. The algorithm, consisting of a spatial (multi‐atlas) and an appearance (random forest classifier) model, was trained using 30 randomly chosen 3DGRASS MRIs, of which the left knees were manually segmented using ITK‐SNAP [36]. For the multi‐atlas part of the algorithm, the Elastix toolbox [37] was used to register the manually segmented (atlas) images to the unseen (target) images. The obtained registration parameters were applied to the manually segmented atlas labels. The probability of a voxel in the target image being part of the knee bones was calculated by averaging the deformed labels of all the atlas images. The appearance model, a random forest classifier, was trained on the images' original intensity values and Gaussian scale space features using the 30 manually segmented MRIs [33]. The classifier was then used to classify voxels in the unseen (target) images to be patella, femur, tibia, or background. The probabilities of the multi‐atlas and appearance components were combined to obtain the final output of the algorithm.

All right knee images were mirrored using the libraries NiBabel and NumPy [38, 39] in Python (Python Software Foundation, https://python.org/) to enable automatic segmentation with our algorithm, which was trained on left knee MRIs only. After the trained algorithm was applied to all target images, all the segmentations were visually inspected and manually corrected by one researcher (R.P.). Manual corrections included adding and removing small parts that did not require a significant amount of time, such as removing a small bump that sometimes occurred when a part of the infrapatellar fat pad was incorrectly segmented as the patella. Knees were fully excluded if both bone segmentations (patella and femur) consisted of large segmentation errors. Knees were partially excluded when only one of the segmented bones (patella or femur) consisted of large errors. Large segmentation errors were not manually corrected due to time limitations. Poor image quality was the main reason for these large segmentation errors. Bipartite patellae were excluded from the final data set because this anatomical variation caused problems for SSM since these patella models could consist of multiple segmented bone fragments.

The Marching Cube algorithm [40] was applied to the segmentation files to extract three‐dimensional surface models for all the patellae and femora separately using the VTK and SimpleITK libraries [41, 42]. Loose (multiple) bodies were filtered out, and smoothing and re‐meshing were applied to all the 3D surface models using the Python library pyMeshlab [43]. Remeshing was done using the “meshing_isotropic_explicit_remeshing” filter with the minimum angle between faces set to 120 degrees to remove any problematic vertices, followed by Laplacian smoothing (“laplacian_smooth”) with five iterations. These steps were both performed twice to obtain the final meshes. All femora were rigidly registered to a reference shape using iterative closest point matching (ICP) from the Trimesh library [44]. This reference shape was selected based on orientation in the MRI coordinate system, with the femoral shaft aligned to the y axis. The bounding box was determined, and all femora were cut at 7 cm from the bottom of the bounding box in the direction of the shaft using the Python library Trimesh.

Leave‐one‐out cross‐validation and Dice similarity coefficient (DSC) calculation were performed using the 30 training images to evaluate the performance of the two components of the segmentation algorithm and fine‐tune the alpha parameter controlling the influence of the appearance model in the algorithm. The values of the alpha varied from 0.0 to 1.0 with an increment of 0.1. The Dice coefficient ranges between 0 and 1, with a higher coefficient indicating better segmentation results [45, 46].

### 2.4 Statistical Shape Modeling

Two SSMs, one for the patella and one for the femur, were built based on the remeshed bone samples using the in‐house built script as described by Eijkenboom et al. [19]. The same script was used to enhance comparability since different SSM techniques are known to identify different shape modes, even when using the same data set [47]. With a different data set, there will be differences in shape modes, but in this way, these differences will not be due to the method used.

First, all 3D patellae (or femora) were registered using an unbiased registration algorithm [48], minimizing differences in position and orientation. No scaling was applied since differences in size in our young adolescent population were also of interest. After registration, corresponding points across all the bone samples (patellae or femora) were automatically determined using a closest point algorithm. The mean patellar (or femoral) shape was calculated, and the main patellar (or femoral) shape variations were extracted by performing Principal Component Analysis (PCA). One researcher (R.P.) interpreted shape variations explained by each shape mode using an in‐house built 3D viewer and using distance maps that quantify the extent of shape changes from the mean (in mm) at two extremes (i.e., ±3 SD shape deviation from the mean shape). An experienced orthopedic surgeon (T.P.) checked the descriptions of the shape variations using the same in‐house built 3D viewer.

### 2.5 Statistical Methods/Analyses

Descriptive statistics were used to assess participant characteristics. Differences in descriptive statistics between boys and girls were tested using t‐tests for numeric variables and chi‐squared tests for categorical variables. The level of statistical significance for the descriptive statistics was set at *p* < 0.05. Generalized estimated equation (GEE) modeling was used, taking the correlation between two knees within a subject into account, to test the association between the patella and femoral shape modes, BMI‐SDS, and activity measures (sports participation, sports participation at 9 and 13 years old, physical activity, and active transport). To enhance interpretability of the results, only shape modes that explained at least 1% of the total population variation were included in statistical analyses. All GEE analyses were adjusted for sex and age. Bonferroni correction for multiple testing was applied, resulting in different thresholds for significance for patella and femur analyses based on the number of included shape modes (*p* < 0.05/(N‐included shape modes)) [49]. The results were presented as regression coefficients (Betas (β)) with 95% confidence intervals (CI). All the analyses were performed using R (version 4.3.2, R Core Team, 2022).

## 3 Results

**Figure 1:**
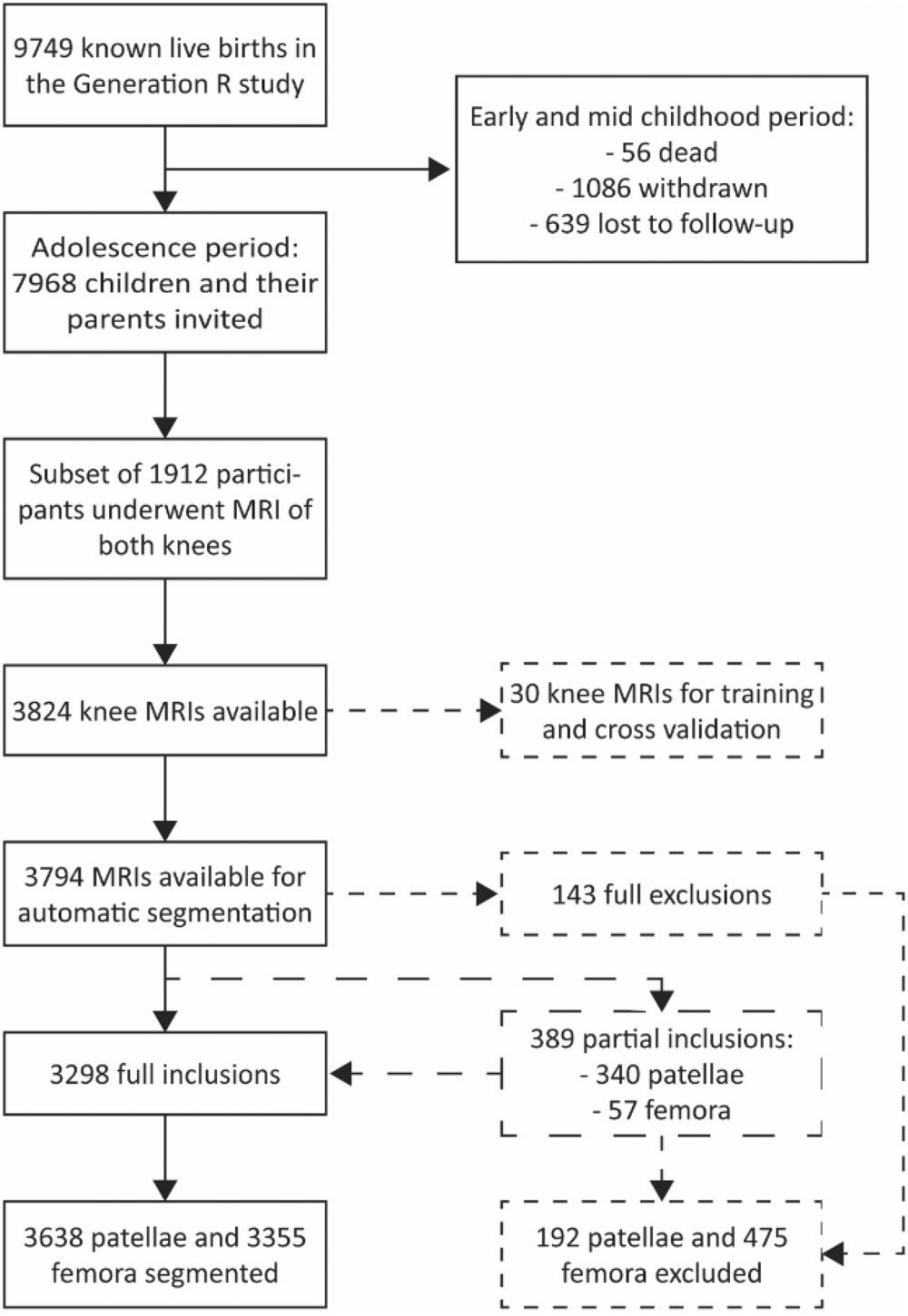
Flowchart on data inclusion and segmentation.

Our study included 1912 young adolescent participants from the Generation R study (see Figure 1). Mean (SD) age was 14.1 (0.67) years and of 52% were girls. All participant characteristics are shown in Table 1. More girls (*N* = 290, 29%) reported not spending any time gaming compared to boys (*N* = 57, 6%). Girls more often reported longer durations (> 2 h) on the computer (34% vs. 26%) or behind the TV (12% vs. 8%) than boys. Boys were taller than girls (166 (8.7) cm vs. 163 (7.0) cm). The children included were physically active for 5.3 (1.8) days overall, with boys reporting slightly more days of physical activity than girls (5.5 (1.8) days vs. 5.2 (1.9) days) (Table 1). 1338 children participated in sports, with football (*n* = 321; 16.8%) and field hockey (*n* = 277; 14.5%) being the most popular, followed by dancing (*n* = 131; 6.9%), judo (*n* = 119; 6.2%), tennis (*n* = 79; 4.1%), athletics (*n* = 65; 3.4%), horseback riding (*n* = 53; 2.8%), swimming (*n* = 26; 1.4%), volleyball (*n* = 25; 1.3%), gymnastics (*n* = 23; 1.2%), skating (*n* = 18; 0.9%), baseball/softball (*n*= 15; 0.8%), korfball (*n* = 15; 0.8%), rugby (*n* = 11; 0.6%), cycling/mountain biking (*n* = 11; 0.6%), and other types of sport (*n* = 149; 7.8%).

Mean (range, SD) DSC values indicating segmentation accuracy in the cross‐validation experiment were high: 0.91 ([0.52 – 0.95], 0.08) for the patella and 0.97 ([0.93‐0.98], 0.01) for the femur. One of the training images was excluded from the final algorithm because of poor segmentation performance (patellar DSC of 0.52). The optimal value for down‐weighting the appearance component before combining it with the multi‐atlas model was determined at a power of 0.4. The algorithm was applied on (1882 left and 1912 mirrored right) 3794 MRIs. Twenty‐one bipartite patellae were excluded, and after visual inspection of all segmentations, 3638 patella samples of 1877 participants and 3355 femur samples of 1811 participants were retained (see Figure 1).

**Table 1:** Participant characteristics (*N* = 1912), overall and separately for boys and girls within the Generation R population. Potential differences between boys and girls were evaluated using t‐tests (continuous variables) and chi‐squared tests (categorical variables).

*Physical factors*	Total (*N* = 1912)	Boys (*n* = 918)	Girls (*n* = 994)	*p*‐values
**Age (years)**	14.1 (0.67)	14.1 (0.65)	14.1 (0.68)	0.866
**BMI‐SDS** 1	0.43 (1.2)	0.38 (1.2)	0.47 (1.2)	0.095
**Weight status:**				0.656
Underweight	216 (11%)	109 (12%)	107 (11%)	
Normal weight	1390 (73%)	667 (73%)	723 (73%)	
Overweight and obese	304 (16%)	141 (15%)	163 (16%)	
**Height [cm]** 1	164 (7.9)	166 (8.7)	163 (7.0)	**< 0.001**
***Sedentary behavior***				
**Hours of gaming per weekday** 2				
No gaming	347 (18%)	57 (6%)	290 (29%)	**< 0.001**
Less than 1 h	346 (18%)	140 (15%)	206 (21%)	
1‐2 h	414 (22%)	281 (31%)	133 (13%)	
> 2 h	267 (14%)	164 (18%)	103 (10%)	
**Hours of computer usage per weekday** 3				
No computer	83 (4%)	51 (6%)	32 (3%)	**< 0.001**
Less than 1 h	303 (16%)	171 (19%)	132 (13%)	
1‐2 h	413 (22%)	181 (20%)	232 (23%)	
> 2 h	576 (30%)	240 (26%)	336 (34%)	
**Hours of TV per weekday** 4				
No TV	210 (11%)	106 (12%)	104 (10%)	**< 0.001**
Less than 1 h	570 (30%)	294 (32%)	276 (28%)	
1‐2 h	402 (21%)	172 (19%)	230 (23%)	
> 2 h	188 (10%)	69 (8%)	119 (12%)	
***Physical activity behaviors***				
**Sports participation at 13 years** 5, Yes	1338 (70%)	657 (72%)	681 (69%)	**0.038**
Only at 13 years old	461 (24%)	227 (25%)	234 (24%)	0.933
Only at 9 years old	100 (5%)	51 (6%)	49 (5%)	
Both at 9 and 13 years old	877 (46%)	430 (47%)	447 (45%)	
**Physical activity (N days/week active for at least 1 h)** 6	5.3 (1.8)	5.5 (1.8)	5.2 (1.9)	**0.004**
**Active transportation** 7, ≥ 1 trip/week, yes	1176 (62%)	550 (60%)	626 (63%)	0.945

*Note:* All values are given as mean (standard deviation) or *n* (%).

^1^ missing *N* = 2.

^2^ missing *N* = 538.

^3^ missing *N* = 537.

^4^ missing *N* = 542.

^5^ missing *N* = 327.

^6^ missing *N* = 539.

^7^ missing *N* = 582.

**Figure 2:**
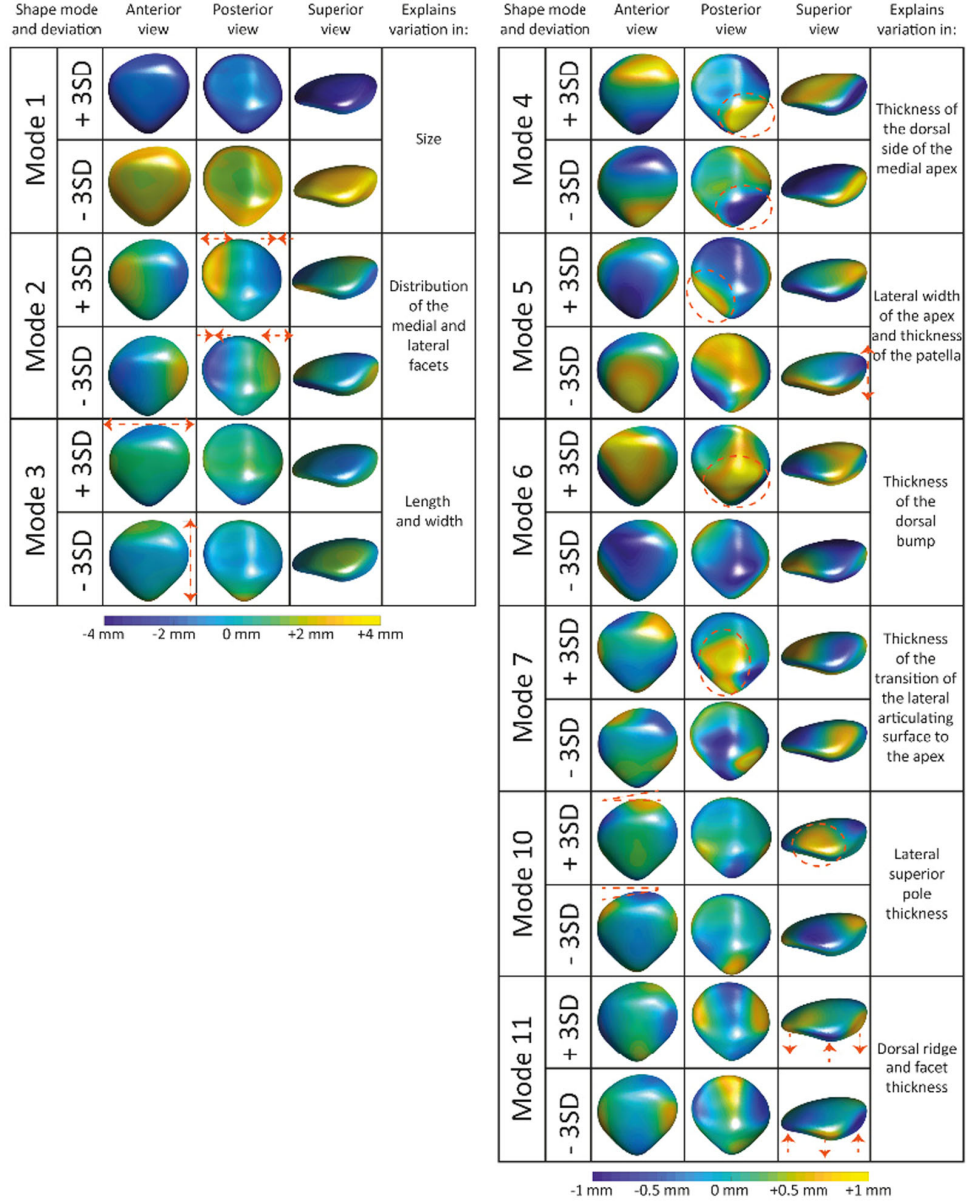
Distance maps of the patella shape modes (1‐7, 10 and 11) showing the distance (in millimeter) from the mean in the ±3 SD direction projected on the mean shape. The distance on the left side ranges from −4 to +4 mm and on the right ranges from −1 to +1 mm.

**Figure 3:**
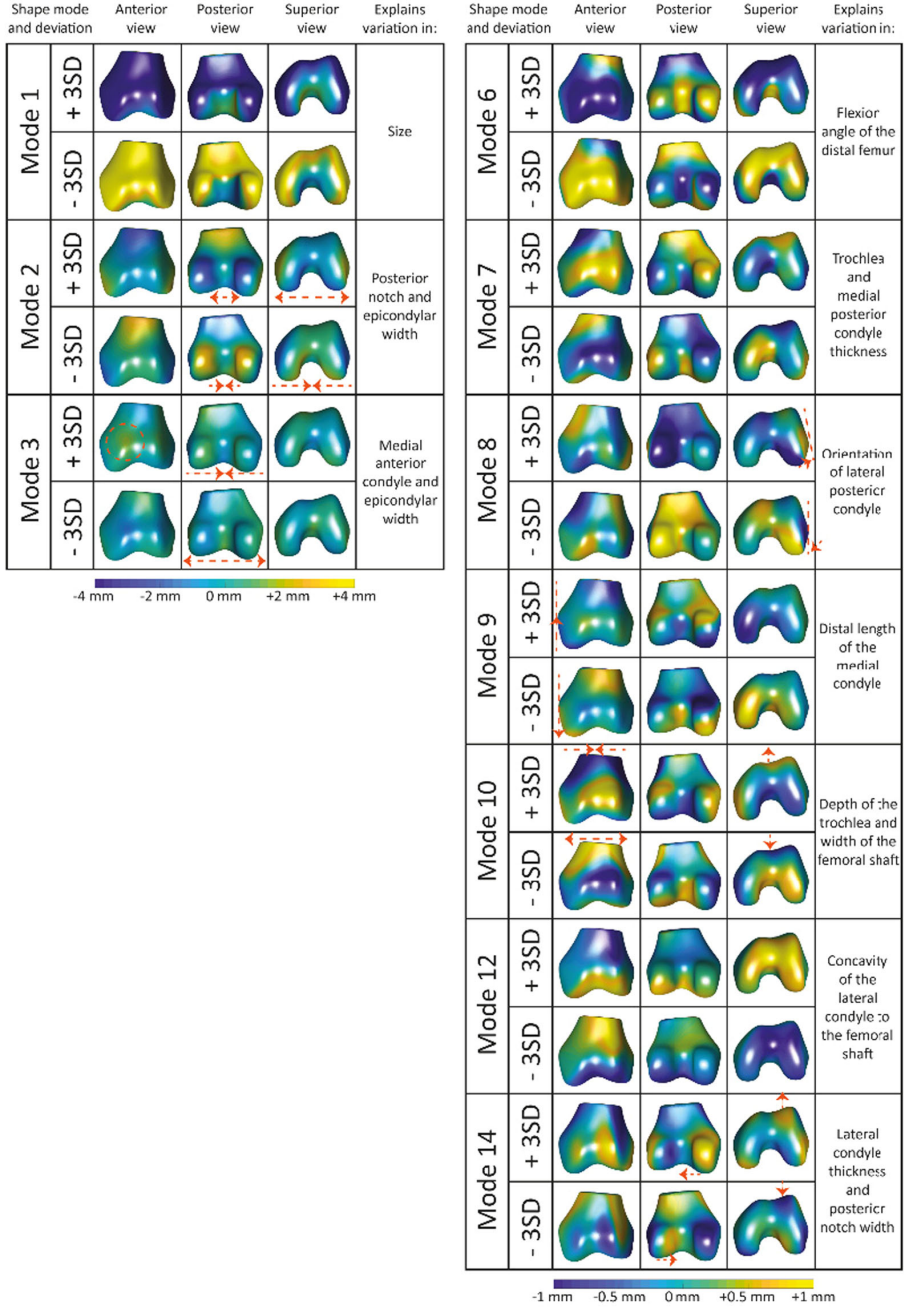
Distance maps of the femoral shape modes (1‐3, 6‐10, 12 and 14) showing the distance (in millimeter) from the mean in the ±3 SD direction projected on the mean shape. The distance on the left side ranges from −4 to +4 mm and on the right ranges from −1 to +1 mm.

The SSMs for the patella and femur were constructed separately. The first eleven patella shape modes and the first fourteen femoral shape modes were included in the statistical analyses, resulting in the Bonferroni adjusted significance thresholds of *p* < 0.0045 and *p* < 0.0036, respectively (Tables 2 and 3). The distance maps presented in Figures 2 and 3 illustrate the patella and femur shape variations in our study population. Shape modes 1 of the patella and femur described 44% and 48% of the total shape variations, respectively. Both modes primarily reflected variations in size and were correlated with stature.

**Table 2:** Associations between loading features and patellar shape modes (Betas with 95% confidence interval).

Mode	Explained variance	BMI‐SDS	Sports at 13^1^	Sport at 9 & 13^2^	Physical activity^3^	Active transportation^4^
1	44.4%	**−0.133 [−0.166; −0.100]** a ^,^ b	**−0.308 [−0.426; −0.190]** a ^,^ b	−0.090 [−0.185; 0.004]^a^^,^ b	**−0.040 [−0.064; −0.015]** a ^,^ b	**−0.323 [−0.468; −0.177]** a ^,^ b
2	16.1%	**0.174 [0.141; 0.208]** a	−0.006 [−0.122; 0.109]^a^	−0.089 [−0.186; 0.008]^a^	0.019 [−0.005; 0.042]^a^	−0.071 [−0.215; 0.073]^a^
3	7.0%	**0.370 [0.338; 0.403]** a ^,^ b	−0.012 [−0.143; 0.120]^a^^,^ b	−0.120 [−0.228; −0.013]^a^^,^ b	−0.031 [−0.058; −0.004]^a^^,^ b	−0.201 [−0.366; −0.037]^a^^,^ b
4	5.0%	**0.068 [0.037; 0.099]** a ^,^ b	0.064 [−0.051; 0.178]^a^^,^ b	−0.048 [−0.144; 0.048]^a^^,^ b	−0.001 [−0.026; 0.023]^a^^,^ b	0.018 [−0.139; 0.176]^a^^,^ b
5	3.5%	**−0.119 [−0.153; −0.086]** b	0.030 [−0.090; 0.150]	0.014 [−0.088; 0.115]	0.016 [−0.010; 0.042]	0.038 [−0.115; 0.191]
6	3.1%	**0.137 [0.102; 0.173]** a	−0.101 [−0.222; 0.020]^a^	−0.059 [−0.163; 0.045]^a^	−0.031 [−0.057; −0.004]^a^	0.004 [−0.151; 0.158]^a^
7	2.2%	**0.108 [0.072; 0.144]** a	−0.038 [−0.156; 0.080]^a^	−0.033 [−0.135; 0.069]^a^	−0.017 [−0.043; 0.009]^a^	−0.010 [−0.162; 0.142]^a^
8	1.8%	0.041 [0.009; 0.072]^a^	−0.096 [−0.209; 0.017]^a^	0.003 [−0.091; 0.097]^a^	−0.007 [−0.030; 0.017]^a^	0.039 [−0.103; 0.181]
9	1.5%	−0.001 [−0.032; 0.029]^a^	0.114 [0.005; 0.223]^a^	−0.001 [−0.092; 0.091]^a^	0.015 [−0.007; 0.038]^a^^,^ b	0.159 [0.031; 0.288]^a^^,^ b
10	1.3%	**−0.063 [−0.097; −0.030]** a	0.025 [−0.096; 0.146]^a^	0.069 [−0.026; 0.165]^a^	0.005 [−0.020; 0.030]^a^	0.163 [0.004; 0.322]^a^
11	1.1%	**−0.092 [−0.125; −0.059]** b	0.062 [−0.059; 0.183]^a^^,^ b	0.066 [−0.026; 0.159]^a^^,^ b	0.007 [−0.020; 0.034]^a^	0.100 [−0.034; 0.234]^a^

*Note:* All analyses were performed corrected for sex and age. *p* < 0.0045 (Bonferroni adjusted threshold for significance) are shown bold.

^a^ Sex significantly associated with outcome (*p* < 0.05).

^b^ Age significantly associated with outcome (*p* < 0.05).

^1^ All participants that participated in sports at 13 years (*N* = 1338), reference category was no sports participation at 13 (*N* = 247).

^2^ *N* = 877 participants that participated in sports at both 9 and 13 years old. Reference category was (*N* = 461) children participating in sports at 13 only.

^3^ Number of days a week (0–7 days) of being physically active for at least 1 h.

^4^ Active transport, at least one trip to or from school by foot or bike (*N* = 1176). The reference category was no active transport (*N* = 154).

**Table 3:** The associations between loading features and femoral shape modes (Betas with 95% confidence interval).

Mode	Explained variation	BMI‐SDS	Sports at 13^1^	Sport at 9 & 13^2^	Physical activity^3^	Active transportation^4^
1	48.1%	**−0.254 [−0.285; −0.222]** a ^,^ b	**−0.279 [−0.388; −0.169]** a ^,^ b	−0.055 [−0.151; 0.042]^a^^,^ b	**−0.038 [−0.062; −0.014]** a ^,^ b	**−0.264 [−0.413; −0.115]** a ^,^ b
2	7.6%	**−0.048 [−0.077; −0.018]** a ^,^ b	−0.085 [−0.184; 0.014]^a^^,^ b	−0.047 [−0.135; 0.041]^a^^,^ b	−0.009 [−0.030; 0.011]^a^^,^ b	−0.007 [−0.132; 0.118]^a^^,^ b
3	5.2%	**0.059 [0.027; 0.090]** a ^,^ b	−0.049 [−0.159; 0.061]^a^^,^ b	−0.087 [−0.179; 0.005]^a^^,^ b	−0.013 [−0.037; 0.011]^a^^,^ b	−0.025 [−0.157; 0.107]^a^^,^ b
4	4.8%	−0.019 [−0.051; 0.012]^a^^,^ b	−0.059 [−0.164; 0.046]^a^^,^ b	−0.025 [−0.116; 0.066]^a^^,^ b	−0.008 [−0.032; 0.016]^a^^,^ b	0.005 [−0.125; 0.135]^a^^,^ b
5	3.5%	−0.043 [−0.076; −0.011]^a^	0.172 [0.054; 0.290]^a^	−0.034 [−0.128; 0.059]^a^	0.005 [−0.020; 0.030]^a^	0.087 [−0.064; 0.237]^a^
6	2.7%	**0.070 [0.039; 0.100]** a	**−0.177 [−0.281; −0.073]** a	−0.133 [−0.222; −0.044]^a^	**−0.035 [−0.058; −0.012]** a	−0.208 [−0.350; −0.067]^a^
7	2.3%	**0.066 [0.035; 0.097]** a ^,^ b	−0.065 [−0.168; 0.038]^a^^,^ b	0.030 [−0.061; 0.120]^a^^,^ b	−0.025 [−0.048; −0.002]^a^^,^ b	−0.072 [−0.198; 0.054]^a^^,^ b
8	2.0%	**−0.113 [−0.145; −0.080]** a	−0.034 [−0.145; 0.077]^a^	0.020 [−0.073; 0.112]^a^	−0.002 [−0.026; 0.021]^a^	−0.016 [−0.153; 0.120]^a^
9	1.9%	**−0.058 [−0.087; −0.029]**	−0.015 [−0.118; 0.087]	0.085 [−0.005; 0.176]	−0.001 [−0.025; 0.023]	0.132 [0.005; 0.259]^a^
10	1.7%	**0.059 [0.025; 0.093]** a ^,^ b	−0.107 [−0.232; 0.018]^a^^,^ b	−0.058 [−0.157; 0.040]^a^^,^ b	−0.036 [−0.062; −0.009]^a^^,^ b	0.014 [−0.133; 0.161]^b^
11	1.5%	−0.041 [−0.074; −0.008]^b^	0.066 [−0.045; 0.177]	0.096 [0.004; 0.188]	0.012 [−0.011; 0.036]	0.172 [0.032; 0.312]
12	1.3%	−0.046 [−0.077; −0.015]^a^	0.074 [−0.030; 0.178]^a^	**0.141 [0.049; 0.232]** a	−0.005 [−0.028; 0.018]^a^	0.142 [0.011; 0.272]^a^
13	1.1%	0.025 [−0.006; 0.056]^a^	−0.041 [−0.148; 0.066]^a^	−0.039 [−0.132; 0.053]^a^	0.022 [−0.001; 0.045]^a^	0.106 [−0.032; 0.244]^a^
14	1.0%	**−0.063 [−0.095; −0.030]** b	−0.066 [−0.190; 0.057]^b^	0.017 [−0.078; 0.113]^b^	−0.003 [−0.027; 0.020]^b^	0.154 [0.012; 0.296]^b^

*Note:* All analyses were performed corrected for sex and age. *p* < 0.0036 (Bonferroni adjusted threshold for significance) are shown bold.

^a^ Sex significantly associated with outcome (*p* < 0.05).

^b^ Age significantly associated with outcome (*p* < 0.05).

^1^ All participants that participated in sports at 13 years (*N* = 1338), reference category was no sports participation at 13 (*N* = 247).

^2^ *N* = 877 participants that participated in sports at both 9 and 13 years old. Reference category was (*N* = 461) children participating in sports at 13 only.

^3^ Number of days a week (0–7 days) of being physically active for at least 1 h.

^4^ Active transport, at least one trip to or from school by foot or bike (*N* = 1176). The reference category was no active transport (*N* = 154).

GEE analysis revealed that BMI‐SDS was statistically significantly associated with nine patella (modes 1, 2, 3, 4, 5, 6, 7, 10, and 11) and nine femoral (modes 1, 2, 3, 6, 7, 8, 9, 10, and 14) shape modes (Tables 2 and 3). The patella shape modes associated with BMI‐SDS described variation in size (mode 1), distribution of the medial and lateral facets (mode 2), length and width (mode 3), thickness of the dorsal side of the medial apex (mode 4), lateral width of the apex and patellar thickness (mode 5), thickness of the dorsal bump (mode 6), transition of the lateral articulating surface to the apex (mode 7), lateral superior pole thickness (mode 10), and the proximal dorsal ridge and facet thickness, resulting in a flatter or more curved patellar joint surface (mode 11) (Figure 2). Femoral shape modes associated with BMI‐SDS described variation in size (mode 1), intercondylar notch and epicondylar width (mode 2), medial anterior condyle thicknesses and epicondylar width (mode 3), flexion angle of the distal femur (mode 6), trochlea and posterior medial condyle thickness (mode 7), the orientation of lateral posterior condylar axis (mode 8), the distal length of the medial condyle (mode 9), the depth of the trochlea combined with width of the femoral shaft (mode 10), and lateral condyle thickness and posterior notch width (mode 14) (Figure 3).

Sports participation at 13 years old and a higher level of physical activity were significantly associated with lower SD values in patella shape mode 1 (Table 2), and femoral shape modes 1 and 6 (Table 3), showing a bigger patella, bigger femur, and more flexion of the femoral condyles relatively to the femoral shaft, respectively. Sports participation at both 9 and 13 years old was significantly associated with higher values in femoral shape mode 12 (Table 3), representing a more concave transition of the lateral anterior condyle into the femoral shaft. Active transportation to and from school was associated with lower SD values in shape mode 1 of both patella and femur (Tables 2 and 3), indicating participants that walked or cycled to school at least once a week have bigger patellae and femora.

## 4 Discussion

We developed SSMs of the patella and femur in a young adolescent population. Statistically significant associations between (nine out of eleven) patellar and (nine out of fourteen) femoral modes of shape variation and BMI were found. Similarly, we found associations between (one out of eleven) patellae and (three out of fourteen) femoral modes of shape variations and sports participation, indicating that BMI and sports participation are of importance to bone shape formation and associated with shape variation in the young adolescent population.

Eleven patellar and fourteen femoral shape modes were included in the analysis, explaining 87% and 85% of the total shape variation in the studied population, respectively. The patellar shape variations showed more easily interpretable variations (length, width, thickness) than the femur shape modes, which is due to the complexity of the femur, including multiple axes and surfaces. For example, patella shape mode 3 showed variation in the width and length of the patella, with the −3SD being narrow and long and the +3 SD being wide and short. However, variations in the femoral shape include multiple axes. For example, femoral shape mode 2 corresponds to variations in the trochlear groove's depth and the posterior condyles' width. Interpreting the femoral shape modes is more challenging because of the complex variation of each femoral shape mode.

Children and young adolescents with obesity have higher bone mineral density than their peers [50] and are more likely to experience musculoskeletal symptoms and complaints [51]. In addition to the metabolic effects of obesity on bone mass, excess weight carried in participants with obesity puts additional stress on bones and joints [51], potentially leading to altered bone shape and pain. Our findings seem to support this, as nine patellar and nine femoral shape modes were associated with BMI. From our results, it is clear that the association between shape and BMI is in multiple axes. These shape variations might lead to maltracking or increased joint stress. For example, a thicker dorsal bump of the patella (mode 6) with a shallower trochlear groove (mode 10) could result in a less congruent joint and lead to increased joint stress. Early identification of these changes could inform preventive strategies and give more insight into the impact of obesity on the growing child. Further research has to be done to understand the exact influence of these shapes on knee health.

Furthermore, increased biomechanical loading experienced by the bone might alter the rate of bone turnover in young adolescents. There are signs that bone mineral density is higher in adolescents participating in sports [52, 53]. Additionally, there is evidence in young soccer (compared to non‐athletes) and young tennis players (dominant arm vs. nondominant arm) that bone geometry changes during (pre‐)puberty [5, 20]. Agricola et al. showed that cam lesions of the hip were more prevalent in young active football players than in the control group [20], suggesting that high‐impact sport at a young age, especially around the time of growth plate closure, could be an important factor in the development of bone shape variations and deviations [54]. The current results show that three shape modes are associated with sports participation (patellar shape mode 1 as well as femoral shape mode 1 and 6). The effects of BMI on bone shape were more prominent than the effects of physical activity and sports participation, suggesting that sports participation may contribute less to shape formation than BMI. Another explanation could be that our data provides more detailed information on BMI (continuous) as compared to sports participation (yes or no) and physical activity (number of days, at least 1 h). Alternatively, increased body mass can lead to elevated joint loading during routine activities such as walking and standing [55]. Therefore, the relationship between joint shape and BMI‐SDS likely reflects both passive mechanical influences and individual variability in physical activity rather than being solely attributable to extremes in loading or obesity.

In the hip joint, there is evidence that cam morphology develops gradually over time [54]. Therefore, we aimed to evaluate prolonged exposure to sports by creating a variable combining sport participation at 9 and 13 years old. Femoral shape mode 12, explaining variation in the concavity of the transition from the lateral condyle into the femoral shaft, was associated with the combined sports participation variable, indicating that a longer duration of loading might lead to a more concave femur due to increased stress of the patella and attached muscles on the femoral bone. While this shape mode was significantly associated with prolonged sports participation, it should be noted that the lower shape modes, such as femur mode 12, account for a small portion of the total population variation and show (sub)millimeter differences which may fall within the limits of segmentation accuracy and should therefore be interpreted with caution.

Most studies applying SSM to the knee focus on the tibiofemoral joint in older participants with OA [15], while only a few studies have developed/built an SSM of the patella [13, 19]. Our current study focused on healthy young adolescents, while previous patella SSM studies focused on specific patient populations. Liao et al. studied longitudinal changes in OA features and their association with patellar and femoral bone shape and found a few shape modes present in this population that are comparable to our current study population [13]. In particular, the shape mode explaining changes in intercondylar notch width is similar to femoral shape mode 2 in our study. Liao et al. additionally found an association between a shallower trochlear groove and PFJ degeneration [13]. We found multiple shape modes explaining the depth and thickness of the trochlea (modes 6, 7, and 10). Therefore, it would be interesting to evaluate follow‐up data in our current study to identify which participants will develop pain or even OA later in life. Furthermore, Eijkenboom et al. (2021 and 2023) studied the shape of the patella in two populations: 14‐40‐year‐old PFP and control participants and an older population of women with PFOA and healthy control participants [19, 56]. Since PFP arises during (young) adolescence, a comparison of the shape modes found by Eijkenboom et al. and the current study seems of particular interest. One shape mode identified by Eijkenboom et al. was associated with structural abnormalities linked to OA (BMLs and minor cartilage defects of the patella) in the 14‐40‐year‐old PFP and control participant population [56]. This shape mode mainly explains variations in the thickness of the dorsal bump of the patella. In our current study, this specific shape variation was captured in patellar shape mode 6, which was associated with higher BMI‐SDS and female sex. This thicker dorsal bump may lead to altered stress on the femoral trochlea and, therefore, potentially lead to joint degeneration. Therefore, this particular shape mode, along with the shape modes representing the depth of the trochlea, are of interest for future research, as the additional stress caused by these specific shape modes might, over time, along with other factors, lead to pain or even OA.

### 4.1 Strengths and Limitations

This is the first study to report shape variations of the patella and distal femur in a large open‐population study among adolescents. The use of this study population and the large sample size enhanced the generalizability of the results to the open population since participants with diverse backgrounds were included. Follow‐up measurements, including MRI, of these subjects at 18 years old were completed at the end of 2024, which will enable us to investigate in future research whether and in which participants' bone shape may change in time. However, some limitations need to be addressed.

A segmentation method combining a multi‐atlas and appearance models had been performed before and proved robust and applicable to large population cohort studies [33, 34, 35, 57, 58]. The manual inspection and adaptation of (minor) segmentation errors could have introduced some bias. To minimize this bias, the process of inspection and adaption was standardized by having one researcher (R.P.) inspect and adapt every segmentation in a random order. Additionally, after post‐processing, all the 3D models were inspected by another researcher (N.T.) for verification of correctness.

To enhance interpretability of the shape modes, we only included modes that explained 1% or more of the total shape variation. This was an arbitrary cut‐off and it could be argued that the cutoff is not sufficient and that it is needed to increase this to (e.g.,) 5% as the lower shape modes show many sub‐millimeter variations. Uncertainties in the segmentations might cause these sub‐millimeter variations, which might be statistically but not clinically relevant. However, we believe that differences found after Bonferroni correction remain meaningful due to the large study population.

MRI was performed with both legs simultaneously, resulting in varying knee positioning. Some knees were fully extended, while others were slightly flexed or endo‐ or exorotated. Two separate statistical shape models were created instead of one due to difficulty in determining a proper constrain for the joint space. Additionally, modeling the patella and femur together would increase the difficulty in interpreting the variation found in the shape modes.

Furthermore, ideally, the sports variables would have been split into types of sports which would have enabled us to assess if participating in certain sports leads to different loading patterns and, thus, different shape modes. This was unfortunately not feasible due to a skewed distribution of sports within the study population. Most children participated in similar types of sports, with 16.8% participating in football and 14.5% in field hockey. Certain sports, such as cycling (0.6%) and swimming (1.4%), were less common. Additionally, we had no information on the number of hours per day or week of sports participation. Ideally, we would have had more information enabling us to study the interaction between sports type, intensity, and body mass on joint and bone shape.

Lastly, there could have been some selection bias in excluding the participants who underwent MRI but had poor imaging or segmentation quality. Although they did not differ in age, the excluded participants were more often boys (61%) and had a significantly lower BMI‐SDS (mean 0.07) score than the included population (0.44).

## 5 Conclusion

This study evaluated the association between patellar and femoral shape variations and BMI and sports participation in young adolescents. Statistically significant associations were found between adolescent BMI and nine patellar and nine femoral shape modes. Additionally, sports participation was significantly associated with one patellar and two femoral shape modes and sports participation for a longer period of time with another femoral shape mode. These findings highlight the critical role of BMI in shaping patellar and femoral morphology during adolescence, with sports participation playing a secondary role. Early identification of at‐risk adolescents may enable interventions to reduce the risk of future knee disorders.

## Author Contributions

Jukka Hirvasniemi and Stefan Klein supervised data segmentation. Nazli Tumer, Amir A. Zadpoor and Edwin H. G. Oei supervised data post‐processing. Nazli Tumer performed statistical shape modeling, Tom M. Piscaer supervised interpretation and correctness of the descriptions of the shape modes. Sita M. A. Bierma‐Zeinstra and Marienke van Middelkoop supervised data analysis and interpretation. Rosemarijn van Paassen performed segmentation, post‐processing, data analysis, interpretation of the results and drafted the manuscript. All authors provided feedback on the manuscript and approved the final version of the manuscript.
